# Books and Authors

**Published:** 1911-10

**Authors:** 


### BOOKS AND AUTHORS

---

**The Long Roll,** by **Mary Johnston;** Houghton, Mifflin & Co., Boston; $1.40. An interesting historic novel of the Civil War, with war maps and illustrations in color.

**Manual of Materia Medica for Medical Students;** by **Dr. E. Quin Thornton,** Assistant Professor of Materia Medica in Jefferson Medical College; Lea & Febiger, Philadelphia and New York. $3.50; 525 pages.

This is one of the few books which dispense, properly, with a table of contents, though there is a full index. The official materia medica is given in bird's eye view, classified into inorganic and organic chemicals, products of the animal kingdom by classes, and vegetable drugs by botanic families. Part I briefly discusses posology, prescription writing, incompatibilities, etc. Part II, more than half the book, consists in an alphabetic discussion of official drugs and chemicals, and it becomes clear that the work is a reasonably full treatise on therapeutics and toxicology. Part III gives a complete list of Galenicals. Dosage, already mentioned in the various parts, is repeated in the form of the U.S.P. table of approximate adult average doses.

The limitation of the scope of the work to official drugs, places it out of competition with various other books but lends special value to its pages. For many physicians, Thornton's book is worth its price simply as an argument in favor of a more elastic Pharmacapoeia, for others, merely for the sake of the classification of drugs and the lists of Galenicals. While it is designed particularly for students, undergraduate and post-graduate, who must learn various details often forgotten in actual practice, it is far above the quiz compend class and is a valuable, practical work for all.

---

**The Practical Medicine Series,** 10 volumes each year, prices ranging from $1.25 to $2.00 per volumes, $10.00 for the entire set. Under the general editorial charge of **Dr. Gustavus P. Head;** The Year Book Publishers, Chicago.

This review of periodical literature, maintains its standard of excellence and is quite freely illustrated. Reference has already been made to some of the volumes. Vol. 4, Gynaecology is edited by Dr. Emilius C. Dudley; and C. von Bachelle. Vol. 5, Obstetrics, by Drs. Joseph B. DeLee and Herbert M. Stowes. It is impracticable to review in detail, a review of literature. Suffice it to say that the busy physician who wishes the gist of medical advances, or the student who cannot spend time to refer to large indexes, wade through a library of journals and sift out the really original contributions to current literature, here finds most of the work ready prepared.

---

**One Hundred Surgical Problems; Dr. James G. Mumford,** Visiting Surgeon to the Massachusetts General Hospital, etc., Boston; Published by **W. M. Leonard,** Boston, in the Case History Series. Three hundred and fifty-four pages, several plates, $3.00.

This series goes back to the clinical style in vogue in the seventeenth century, citing actual cases, arranged in a systematic way, and drawing general conclusions from the evidence presented. While we do not believe that the didactic text book, with its dicta for typic cases, and merely incidental allusions to actual professional experience can be dispensed with, too many of the new books are obviously made to order by a man with previous books on the same subject spread out on tables. From these, he selects scraps, changes their wording and dictates to a stenographer. A few new points are introduced from journals and a few from the author's (?) experience, the publisher does his best with paper, plates and illustrations, and old authorities are sent to the second-hand dealer or the medical library. It is true that the case-history method is slightly prolix, does not conform so well to typic needs as the didactic method, and is somewhat disjointed. But, it presents actual evidence, it is more interesting to read and the lessons contained impress themselves more readily and more permanently on the mind especially for the practitioner, who is thinking of the test by the patient rather than that by an examining board, and, however much theoretic views change and science alters our conceptions of disease, the greater part of such a work is good for all time. The publisher is to be congratulated on having revived an ancient custom and restored it on modern lines. The author has selected and classified, from a large surgical experience, 100 of the kind of cases which make the greatest demand on the skill and judgment of the general practitioner, which confront him with ethical, diagnostic and therapeutic puzzles. Although a surgeon, the author does not always operate but tries sincerely and, we think, successfully, to help the reader in those frequent sources of anxiety——cases in which the advisability of operation hangs in the balance—and in which actual surgical consultation leads, in many instances, to ill-advised and fruitless interference, the odium and regret for which attach to the original attendant.

---

**Hand Book of Suggestive Therapeutics, Applied Hypnotism and Psychic Science, Dr. Henry S. Munro,** Omaha. Published by the C. V. Mosby Co., St. Louis, 3d revised edition, 1911. 8 vo, 409 pages, $4.00.

While we hold very conservative views as to the possibility of influencing organic or even definite "functional" diseases, through impressions on the conscious centers, and as to the existence of genuine "subconscious" centers, it cannot be denied that the mental state of the patient has an important bearing on the progress of diseases of all natures nor that the physician's duty and practical usefulness include the encouragement, discipline and control of persons of unstable nervous equilibrium. Dr. Munro's book is eminently practical and sound, although in implication rather than actual statement, it seems to recommend hypnotism for more general use than many consider justifiable.

